# Prolyl Isomerase Pin1 Regulates Mouse Embryonic Fibroblast Differentiation into Adipose Cells

**DOI:** 10.1371/journal.pone.0031823

**Published:** 2012-03-07

**Authors:** Takafumi Uchida, Kengo Furumai, Tomokazu Fukuda, Hirotada Akiyama, Mika Takezawa, Tomoichiro Asano, Fumihiro Fujimori, Chiyoko Uchida

**Affiliations:** 1 Molecular Enzymology, Department of Molecular Cell Science, Graduate School of Agricultural Science, Tohoku University, Miyagi, Japan; 2 Center for Interdisciplinary Research, Tohoku University, Miyagi, Japan; 3 Department of Animal Production Science, Tohoku University, Miyagi, Japan; 4 Department of Medical Science, Hiroshima University, Hiroshima, Japan; 5 Department of Environmental Education, Tokyo Kasei University, Tokyo, Japan; 6 University Health Center, Ibaraki University, Mito, Japan; Ospedale Pediatrico Bambino Gesu', Italy

## Abstract

**Background:**

A peptidyl prolyl *cis/trans* isomerase, Pin1, regulates insulin signal transduction. Pin1 reduces responses to insulin stimulation by binding CRTC2 (CREB-regulated transcriptional co-activator 2) and PPARγ (peroxisome prolifereator- activated receptor γ), but conversely enhances insulin signaling by binding IRS-1 (insulin receptor substrate-1), Akt kinase, and Smad3. Therefore, it is still unclear whether Pin1 inhibits or enhances adipose cell differentiation.

**Methodology/Principal Findings:**

Pin1^−/−^ and wild-type mice were fed with high fat diets and adipose tissue weight was measured. Compared to wild-type mice, Pin1^−/−^ mice had lower adipose tissue weight, while the weight of other tissues was similar. Mouse embryo fibroblasts (MEFs), prepared from both groups of mice, were induced to differentiate into adipose cells by stimulation with insulin. However, the rate of differentiation of MEFs from Pin1^−/−^ mice was less than that of MEFs from wild-type mice. The rate of insulin-induced MEF cell differentiation in Pin1^−/−^ mice was restored by increasing expression of Pin1. We found that Pin1 binds to phosphoThr172- and phosphoSer271-Pro sites in CREB suppress the activity in COS-7 cells.

**Conclusion and Significance:**

Pin1 enhanced the uptake of triglycerides and the differentiation of MEF cells into adipose cells in response to insulin stimulation. Results of this study suggest that Pin1 down-regulation could be a potential approach in obesity-related dysfunctions, such as high blood pressure, diabetes, non-alcoholic steatohepatitis.

## Introduction

A peptidyl prolyl *cis/trans* isomerase, Pin1, binds to phosphorylated Ser/Thr-Pro motifs in a variety of proteins and catalyzes *cis/trans* isomerization of peptidyl prolyl bonds. When we created Pin1^−/−^ mice, the first phenotype we noted was that these mice weighed lower than wild-type mice [1], [2]. We hypothesized that a delay in cell cycle progression in Pin1^−/−^ mice causes this loss in body weight.

It is known that Pin1 regulates the activities of regulatory molecules involved in insulin signaling, such as PPARγ [3], CRTC2 [4], IRS-1 [5], Akt [6], and Smad3 [7]. Pin1 binds phosphorylated Ser84-Pro of PPARγ to reduce its transcriptional activity [3]. Pin1 also binds phosphorylated Ser136-Pro of CRTC2 and decreases nuclear CRTC-CREB complexes to inhibit the transcriptional activity of CREB [4]. On the other hand, Pin1 binds phosphorylated Ser434-Pro of IRS1 to directly upregulate insulin signal transduction [5]. IRS-1 is critical for adipose cell differentiation [8]. Pin1 stabilizes Akt kinase, a signaling protein that is down-stream of IRS-1 in the insulin signal transduction cascade [6]. Smad3 is a protein that is activated in response to TGFβ. Smad3 interferes with interactions between C/EBPα and PPARγ and inhibits PPARγ activity [9]. Pin1 accelerates degradation of Smad3 and suppresses TGFβ signaling [7], thereby upregulating insulin signaling.

Immature adipose cells cannot store sufficient triglycerides, and we hypothesized that Pin1 promotes triglyceride storage by enhancing the differentiation of immature cells to mature adipose cells. In this report, we demonstrate the biological role of Pin1 in regulating cellular fat storage in response to insulin signaling.

## Results

### The adipose tissue measured by ComputerTomography is lower in Pin1^−/−^ mice

**Figure 1:**
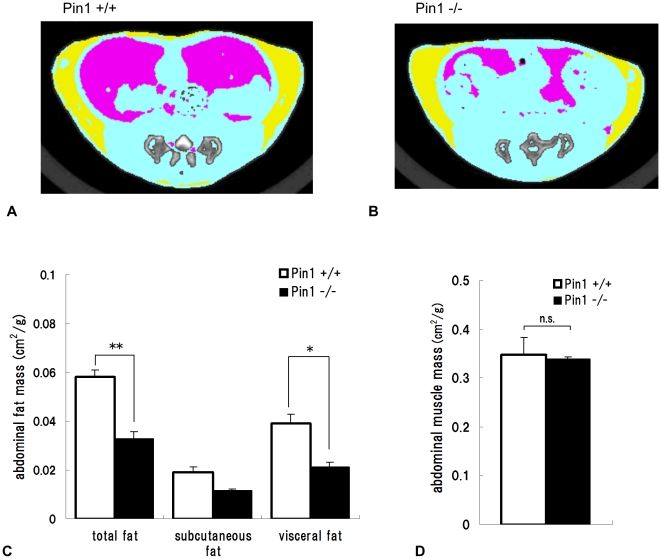
Computed Tomography analysis of wild-type and Pin1^−/−^ mice. The abdomens of 16 week-old male wild-type (A) and Pin1^−/−^ (B) mice were scanned (yellow; subcutaneous fat, pink; visceral fat, blue; muscle). Quantities of subcutaneous, visceral, and total adipose tissues (**p<0.01) (C), and the other tissues, mainly muscle (D), were measured from the images. The areas were measured from the computer tomography images (cm^2^) and they were adjusted for body weights of mice (±SEM, n = 3).

The abdomens of 16 week old male wild-type and Pin1^−/−^ mice were scanned by computer tomography and the images were colored as follows: yellow; subcutaneous fat, pink; visceral fat, blue; muscle (Fig. 1A,B). Quantities of subcutaneous and visceral adipose tissues were determined from the colored areas (Fig. 1C). These results show that the quantity of adipose tissue in Pin1^−/−^ mice was less than that of wild-type mice. The differences in total and visceral fat between wild-type and Pin1^−/−^ mice were significant (Fig. 1C). On the other hand, muscle mass in Pin1^−/−^ and wild-type mice was similar (Fig. 1D).

### The lack of Pin1 leads to lower fat accumulation in high fat diet mice

**Figure 2:**
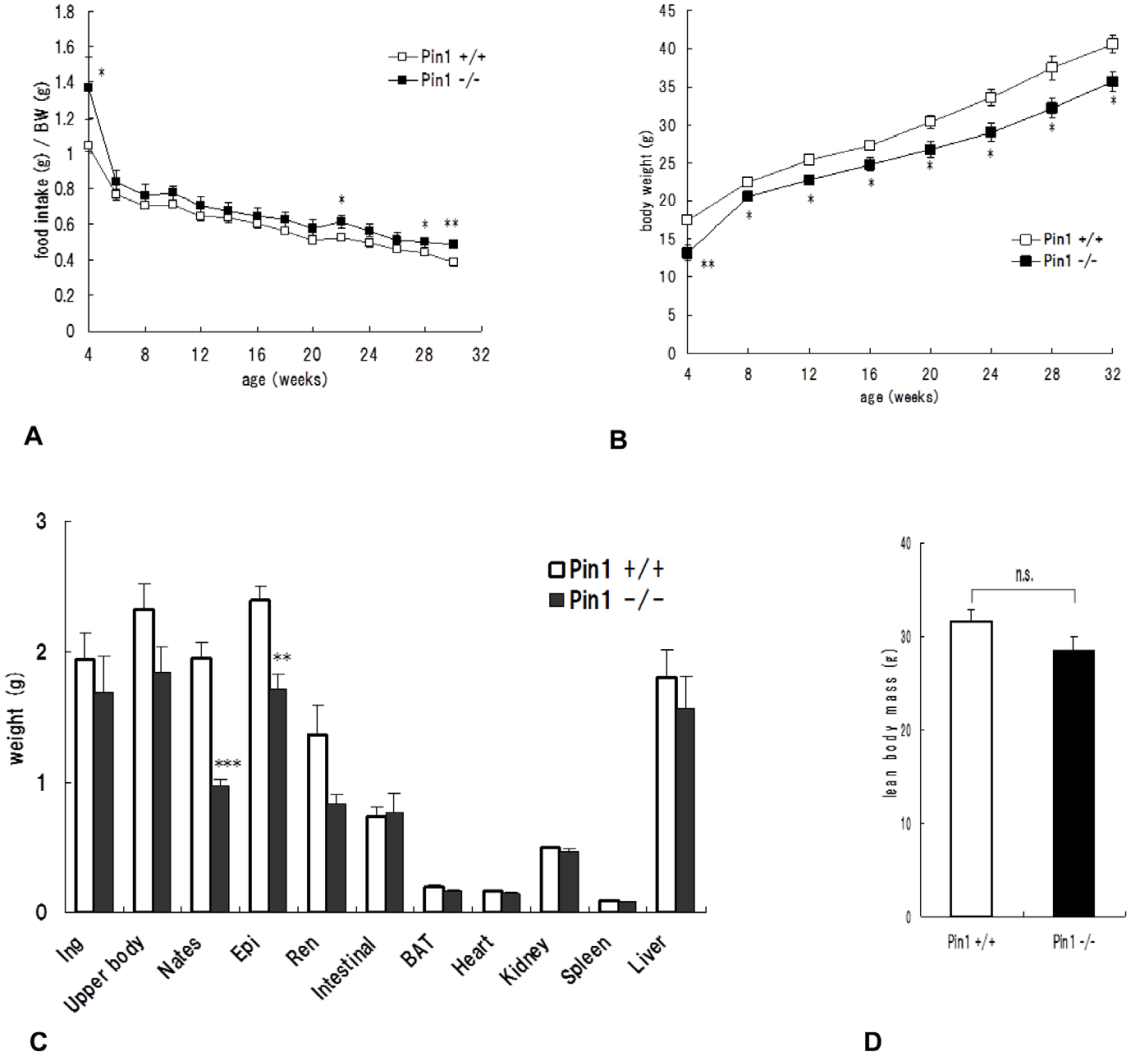
Relationship between quantity of fat and Pin1 expression. High fat diets were fed to wild-type (n = 8) and Pin1^−/−^ (n = 5) mice between the ages of 4–28 weeks and the amount of food intake (A) and body weight (B) were recorded. Weights of subcutaneous and genital fat tissues were measured (C). The total weight of all fat tissues removed was also measured (D). The amount of food intake per mouse weight was monitored. Student t-test *p<0.05, **p<0.01, ***p<0.001.

High fat diets were fed to the mice from 4 to 28 weeks old. Food intake and weight of the mice were monitored. Interestingly, although the intake of food by Pin1^−/−^ mice was greater than that of wild-type mice (Fig. 2A), increases in body weight were lower in knockout animals (Fig. 2B). Mice were then sacrificed and the weights of organs and fat tissues were measured. The weight of each fat tissue, such as buttock fat tissue, which represents subcutaneous fat, and genital fat tissue, which represents visceral fat, from Pin1^−/−^ mice weighed lower than those from wild-type mice (Fig. 2C). On the other hand, the weights of organs, such as heart, kidney, liver, spleen, and brown fat tissues were similar (data not shown). Furthermore, the total weight of removed fat tissues was not different between Pin1^−/−^ and wild-type mice (Fig. 2D).

### Area sizes of mouse adipose cells

**Figure 3:**
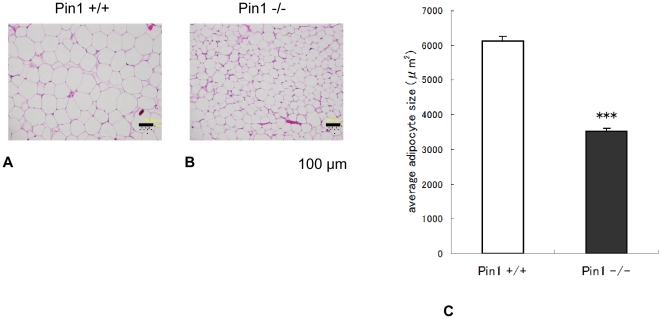
Pathological analysis of adipose tissues from wild-type and Pin1^−/−^ mice. Paraffin-embedded sections of inguinal fat tissue from wild-type (A) and Pin1^−/−^ (B) mice were stained with hematoxylin and eosin. The size of cells was analyzed quantitatively with Image J analysis soft. The average sizes of the adipose cells of wild and Pin1^−/−^ mice were 6129.6±136.0 µm^2^ and 3516.6±87.0 µm^2^ (±SEM) respectively (Figure 3C). Student t-test p<0.001.

Pathological analysis of genital fat tissue revealed that adipose cells from Pin1^−/−^ and wild-type mice are similar (data not shown). However, adipose cells from inguinal fat tissue of Pin1^−/−^ mice were smaller than those from wild-type mice (Fig. 3A, B). We measured the area of cells quantitatively with Image J analysis soft. The average sizes of the adipose cells of wild and Pin1^−/−^ mice were 6129.6±136.0 µm^2^ and 3516.6±87.0 µm^2^ (±SEM) respectively (Fig. 3C) and the difference was statistically significant, p<0.001.

### The inhibition of Pin1 affects adipose-like differentiation in fibroblasts

**Figure 4:**
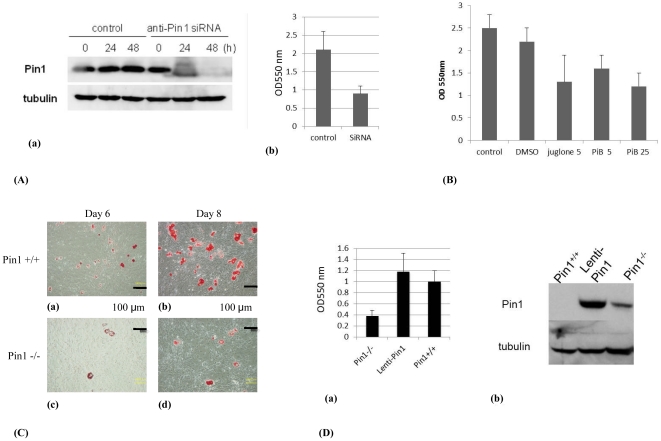
Differentiation of fibroblasts into adipose cells. (A) NIH3T3L1 cells were transfected with 20 µg mock or SiRNA-Pin1 plasmids, cultured for 24 and 48 hours, and the expression levels of Pin1 were examined with western blot analysis (a). The NIH3T3-L1 cells were incubated in DMEM medium, containing; 0.5 mM 3-isobutyl-1-methylxanthine, 1 µM dexamethasone, and 1.7 µM insulin for 48 hours, treated with 4% paraformaldehyde and 60% 2-propanol and then stained with Oil Red O. The amount of Oil Red O extracted from cells was determined by measuring the absorbance at 550 nm (b). (B) NIH3T3-L1 cells were incubated in the same medium with juglone (5 µM) or PiB (5, 25 µM), and the fat was measured with oil red O assay. (C) Wild-type (a, b) and Pin1^−/−^ (c, d) MEFs were stimulated in the same way as NIH3T3 cells for 6 (a, c) and 8 days (b, d), stained with Oil Red O, and analyzed by microscopy. (D) Pin1^−/−^ MEF, Lenti-Pin1 (Pin1^−/−^ MEF-infected with lentiviral *Pin1* cDNA), and wild-type MEF were also examined like (C) (a). Pin1 levels of the Pin1^−/−^ MEFs, Lenti-Pin1^−/−^, and wild-type MEFs were analyzed with western blot (b).

NIH3T3-L1 cells become differentiated into adipose cells containing fat droplets upon stimulation with insulin. The fat droplets were stained red by Oil Red O solution and the rate of differentiation was determined semi-quantitatively. siRNA against Pin1 [4], [10] decreased the Pin1 level in NIH 3T3-L1 cells in 24 hours (Fig. 4A-a) and inhibited their differentiation into adipose cells (Fig. 4A-b). The inhibitory effect of Pin1 depletion was further confirmed by using two Pin1 inhibitors, juglone and PiB (diethyl-1,3,6,8- tetrahydro- 1,3,6,8- tetraoxobenzol - phenanthroline- 2,7-diacetate). Juglone is the most well known inhibitor of Pin1 even if it has the drawback that it blocks transcription directly [11]. Therefore we also used the other Pin1 inhibitor PiB [12]–[14]. 5 µM of juglone inhibited the differentiation as strong as 25 µM of PiB (Fig. 4B). We then generate MEFs from wild-type and Pin1^−/−^ mice that were induced to differentiate into adipose cells by insulin treatment and the fat droplets stained with Oil Red O were observed under a microscope. The wild-type MEFs differentiated into adipose cells that can be clearly observed after 6 days (Fig. 4C-a). 8 days later, differentiation of the cells had progressed further with the number of cells staining red increasing (Fig. 4C-b). Pin1^−/−^ MEFs did not differentiate as much as did wild-type MEFs after 6 days of insulin treatment (Fig. 4C-c), and even after 8 days, the number of stained cells was much less (Fig. 4C-d). Wild-type MEFs, Pin1^−/−^ MEFs-treated with lenti-*Pin1* (Lentiviral vector expressing Pin1), and mock-transfected (Lentiviral vector without insert) Pin1^−/−^ MEFs were stimulated with insulin for 8 days, after which the rate of differentiation of MEFs was determined by measuring the absorbance of red color derived from Oil Red O-stained fat droplets. The intensity of red staining in mock-transfected Pin1^−/−^ MEFs was significantly lower than that of wild-type MEFs. However, transfection of *Pin1* cDNA into Pin1^−/−^ MEFs increased the intensity of red staining to similar, or slightly higher, levels compared to wild-type MEFs (Fig. 4D-a). Levels of Pin1 expressed in MEFs transfected with lenti-Pin1 were higher than those in wild-type MEFs (Fig. 4D-b).

### Pin1 interacts with CREB suppressing its transcriptional activity

**Figure 5:**
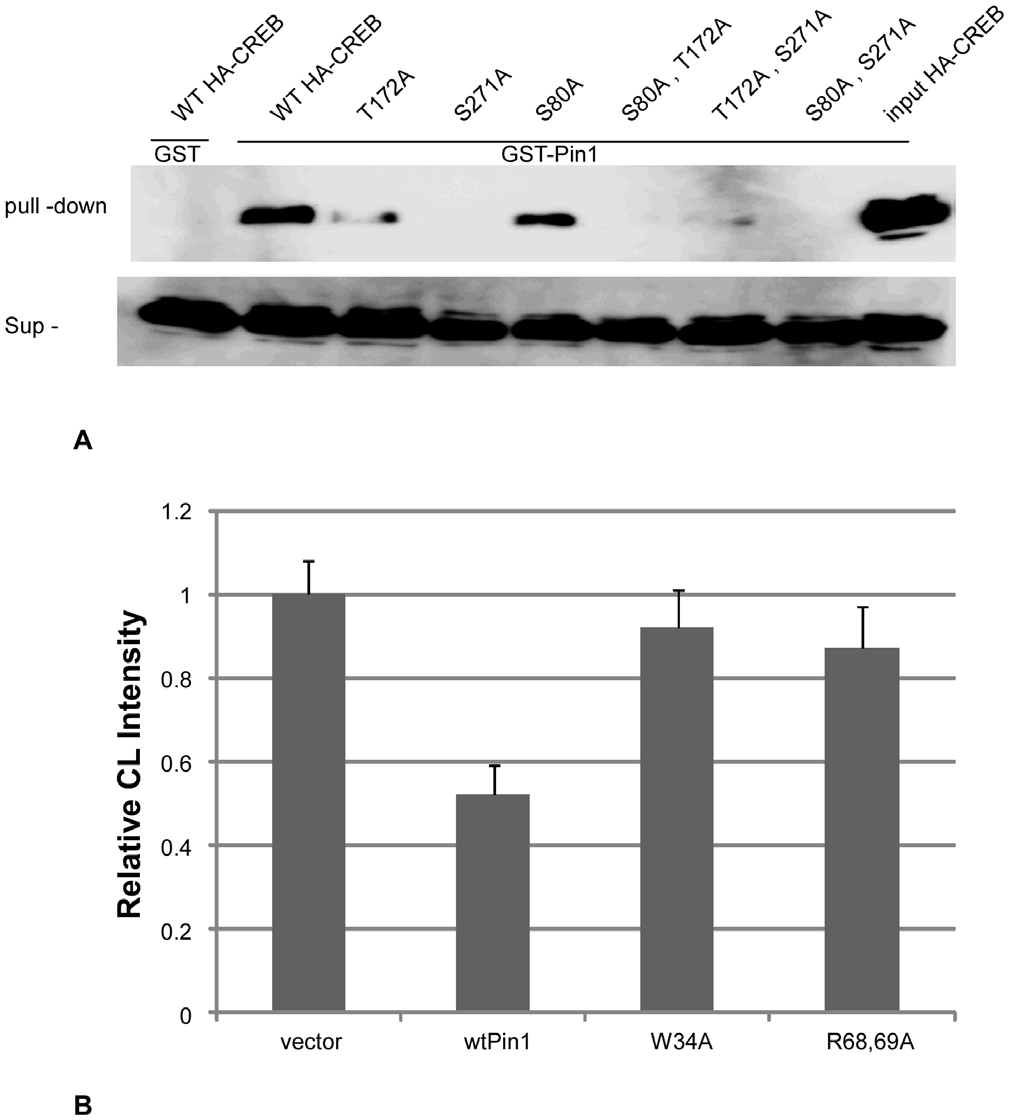
Association of Pin1 and CREB. (A) Lysates of COS-7 cells, transfected with *HA-CREB* (*wild-type; T172A; S271A; S80A; S80A/T172A; T172A/S271A; S80A/S271A*) and stimulated with 10 µM forskolin for 6 hr, were pulled down with GST-Sepharose (control), GST-Pin1-Sepharose, and input (upper panel). CREB levels in the supernatant are shown in the lower panel. (B) 10^6^ of COS-7 were co-transfected with pCRE-Luc and CMV-Pin1 (wt; wild type, W34A; mutation at WW domain, R68,69A; prolyl isomerase mutant). The cells were treated with 100 µM of forskolin (CST) for 6 hours and luciferase activities were determined using the Dual-Luciferase Reporter Assay System (Promega).

Finally, whether Pin1 directly binds to and regulates CREB in MEFs was examined. Pin1 bound CREB directly after stimulation of MEFs with 1 or 10 µM forskolin. There are 3 Ser/Thr-Pro sites in CREB: Ser80, Thr172, and Ser271. Pin1 did not bind to CREB with Ala mutation at Thr172 or Ser271 but bound to CREB with Ala mutation at Ser80 (Fig. 5A). These results suggest that Pin1 directly binds to CREB at phosphoThr172-Pro and phosphoSer271-Pro. Transcriptional assays showed that Pin1 suppresses the transcriptional activity of CREB in COS-7 cells. The Pin1 with the mutation at WW motif or the substrate binding pocket lost the suppressive function (Fig. 5B).

## Discussion

Our computer tomography images suggested that Pin1^−/−^ mice have less fat tissue than wild-type mice. In order to verify this, high-fat diets were fed to these mice, after which various fat tissues were removed and weighed. These results clearly showed that there were significantly less subcutaneous and visceral fat tissues in Pin1^−/−^ mice, suggesting they lose the ability to store lipid in these tissues. In fact, adipose cells from inguinal fat tissue of Pin1^−/−^ mice were smaller than those of wild-type mice. It has been reported that the average diameter of a mature adipose cell is approximately 60 µm. However, once adipose cells aggressively uptake triglycerides, cell size increases (diameter: 100 µm [15]). In our studies, adipose cell area sizes of wild-type and Pin1^−/−^ mice were measured. The area size is more reliable than the diameter because the cell shape is not round. The average adipose cell area sizes of wild-type and Pin1^−/−^ mice clearly show that Pin1 enhances maturation of adipose cells and promotes uptake of lipid.

A fibroblast cell line, NIH3T3L1 differentiates into adipose cells upon insulin treatment. We examined the effect of Pin1 on the differentiation by reducing the expression level of Pin1. SiRNA caused knockdown of Pin1 and inhibited the NIH3T3L1 cell differentiation into adipose cells. In the same way, Pin1 inhibitors, juglone and PiB also blocked the differentiation. Also MEFs can be differentiated into adipose cells by the treatment of the cells with insulin [8]. Results from insulin treatment showed that Pin1^−/−^ MEF differentiated less than wild-type MEFs. However, the rate of differentiation of Pin1^−/−^ MEF into adipose cells was restored to levels similar to that of wild-type MEFs by Pin1 forced expression. These results show that it is possible to regulate fibroblast differentiation into adipose cells by regulating the expression, or activity, of Pin1.

Pin1 is known to bind to CRTC, thereby suppressing CREB activity [4]. We hypothesized that in conditions where CREB is overexpressed, Pin1 might directly bind to CREB and activate it. As expected, Pin1 directly bound CREB at phosphoThr172-Pro and at phosphoSer271-Pro sites. However, as previously reported in HepG2 cells [4], Pin1 suppressed the transcriptional activity of CREB in COS-7 cells. These results suggest that suppression of CREB activity by Pin1, whether by direct or indirect mechanisms, may be a factor underlying enhanced differentiation of fibroblasts to adipose cells in response to insulin stimulation. Further studies are required in order to more fully elucidate mechanisms by which Pin1 enhances differentiation of fibroblasts into adipose cells.

One of the most important functions of adipose tissues is to store triglycerides that can be used when energy is required. Another critical function of adipose tissue is to produce a variety of adipokines that control whole body metabolism [16]. Thus, results of this study suggest that Pin1 down-regulation could be a potential approach in obesity-related dysfunctions, such as high blood pressure, diabetes, non-alcoholic steatohepatitis. Future studies could help for discovering an inhibitor against Pin1 with less toxicity to do animal study and clinical application in future [17]–[19].

## Materials and Methods

### Animal studies

Our study was approved by the Tohoku University Animal Use and Care Committee; approval ID 76-19-84. High fat diets, 20–25 g/week of HFD-60 (Oriental Kobo), were fed to 4-week-old wild-type (n = 8) and Pin1^−/−^ mice (n = 5) [1], with food intake and body weight being measured weekly. High fat diet experiments were continued for 28 weeks.

### Cells

NIH3T3-L1 cells (JCRB9014) were provided by the Japanese Human Science Foundation. MEFs were prepared as previously reported [1], [10]. COS7 and HEK293 were cultured as written in the previous report [20]. We did not use previously unpublished cell lines in this manuscript.

### Statistical analysis

Values are reported as means ± SEM. Statistical analysis of differences between mean values was carried out by Student's *t* tests. Values of p<0.05 were considered to be statistically significant.

### Computer Tomography Analysis

The abdomens of 16 week-old male wild-type and Pin1^−/−^ mice were scanned by Computer Tomography (LCT-100 Lite (Aloka)), and the images were colored as follows: yellow; subcutaneous fat, pink; visceral fat, blue; muscle. Computer Tomography scans were performed at 1.5 mm intervals×20 slices. The areas were measured from the Computer Tomography images (cm^2^) and they were adjusted for body weights of mice (±SEM, n = 3).

### SiRNA plasmid against Pin1

pSilencer™ (Applied Biosystems) was digested with ApaI and EcoRI and annealed oligos *(Pin1:* *5′*GGGCCCGCCGAGTGTACTACTTCAATTCAAGAGATTGAAGTAGTACACCTCGGCTTTTTTGATTC*3′* , control: *5′*GGCCCTCGTATGTTGTGTGGAATTTTCAAGAGAAATTCCACACAACATACGATTTTTTGATTC*3′* ). Transfection of the plasmid (20 µg) was conducted by using Lipofectamine 2000 (Invitrogen) as previously described [20]. After 24 and 48 hours, expression level of Pin1 was examined by western blot.

### Lentiviral- Pin1 cDNA

HEK293 cells were transfected with the packaging plasmids (pCMV-VSVG-RSV-REV, pCAG-HIV-gp), which were kindly provided by Hiroyuki Miyoshi, and Pin1 expression vector (pCDH- CMV-*Pin1*-EF1-copGFP). The supernatant of the culture medium was passed through 0.45 µM filters (Millipore) and used as lentiviral *Pin1* cDNA (4 µg/ml). The lentiviral Pin1 was added into the medium to increase Pin1 expression.

### Differentiation of Cells

NIH3T3-L1 (control and Pin1-knockdown) and MEFs (wild-type and Pin1^−/−^) were incubated in DMEM medium, containing 0.5 mM 3-isobutyl- 1-methylxanthine (Nakalai), 1 µM dexamethasone (Sigma) and 1.7 µM insulin (Wako) for 48 hours. Cells were cultured in insulin-containing medium for 48 hours. Juglone (Sigma) [21] and PiB [12] were solved in ethanol and DMSO respectively (10 mM) and added into the medium to be 5 or 25 µM.

### Histopathology

For histopathological analysis, paraffin-embedded sections were stained with hematoxylin and eosin (Wako). The area of 200 adipose cells in the three different points were measured by using Image J analysis soft [22] and the average areas of the wild and Pin1^−/−^ mouse adipose cells were statistically compared.

### Oil Red O Staining for Cultured Cells

NIH3T3-L1 and MEFs were treated with 4% paraformaldehyde and 60% 2-propanol and then stained with Oil Red O (Sigma). The stained cells were observed with a BZ-8100 microscope (Keyence) and the quantity of Oil Red O- extracted from cells was determined by measuring absorbance at 550 nm with a DTX 880 Multimode Detector (Beckman Coulter).

### Western Blot Analysis

Pin1 was detected using rabbit anti-Pin1 antibodies (Calbiochem), followed by anti-rabbit IgG HRP-linked antibodies (Cell Signaling Technology). α-Tubulin was used as a loading control. Chemiluminescence was detected with an LAS-3000 Image Analyzer (Fuji Film).

### Pull-down Assay

Lysates from COS-7 cells transfected with *HA-CREB* (*wild-type; T172A; S271A; S80A; S80A/T172A; T172A/S271A; S80A/S271A*) were incubated with 30 µg of GST-Pin1 for 30 min at 4°C. Glutathione-sepharose 4B beads (GE Healthcare) were added to the mixture and incubated at 4°C with gentle rocking for a further 1 hr. The beads were precipitated by centrifugation at 3,000× *g* in a microcentrifuge and washed three times with protein buffer. Following the final wash, the beads were resuspended in 25 µl SDS-PAGE sample buffer containing 50 mM DTT. Wild-type and mutant HA-CREBs were blotted onto membranes and were identified with anti-HA antibody (Genscript).

### Transcriptional Assay of CREB

10^6^ of COS-7 cells were co-transfected with pCRE-Luc and CMV-Pin1 (wt; wild type, W34A; mutation at WW domain, R68,69A; prolyl isomerase mutant) (0.25 µg/well), with an internal reporter, pRL-TK. The cells were treated with 100 µM of forskolin (CST) for 6 hours and luciferase activities were determined using the Dual-Luciferase Reporter Assay System (Promega).
